# Synthetic vascular grafts as a new treatment option for space-occupying tumor bed cysts

**DOI:** 10.1007/s00701-022-05123-y

**Published:** 2022-01-25

**Authors:** Simon Schieferdecker, Thomas Beez, Marion Rapp, Daniel Hänggi, Marcel Kamp, Michael Sabel

**Affiliations:** 1 Department of Neurosurgery, Medical Faculty, Heinrich-Heine-University, Moorenstrasse 5, 40225 Düsseldorf, Germany; 2 Department of Neurosurgery, Medical Faculty, Friedrich-Schiller-University, Bachstrasse 18, 07743 Jena, Germany

**Keywords:** Slit valve, Glioma, Vascular grafts, Tumor bed cyst, Shunting

## Abstract

**Introduction:**

Several authors have reported the formation of slit valves as the underlying pathomechanism of space-occupying tumor bed cysts. Iatrogenic slit valves following the resection of high-grade gliomas have been linked to certain risk factors such as intraoperative opening of the ventricles and attempts to seal these. The best therapeutic management of such cystic lesions remains elusive. Several treatment options such as cyst fenestration or cystoperitoneal shunting have been employed but remain associated with high rates of recurrence. With the given complications of the above-described treatment options, the objective was to devise a new therapy option that is safe and effective and treats the slit valve itself rather than its symptoms.

**Methods:**

Between the years of 2010 and 2020, we successfully treated four patients with high-pressure tumor bed cysts following glioma resection by implantation of synthetic ringed vascular grafts into the slit valve.

**Results:**

Postoperatively, the tumor bed cysts were regressive in all patients. Moreover, none of the treatment patients developed any complications associated with the implanted vascular grafts. Revision-free survival was 10, 12, 53, and 126 months, respectively.

**Conclusion:**

The use of synthetic vascular grafts as a means of stenting slit valves is a safe and effective novel treatment option for high-pressure tumor bed cysts.

**Supplementary Information:**

The online version contains supplementary material available at 10.1007/s00701-022-05123-y.

## Introduction

Radical resection with adjuvant radiochemotherapy has been established as the modern standard treatment regime for patients with high-grade glioma (HGG) [9, 20]. A multitude of prognostic factors have been identified that affect the long-term survival of patients with HGG, such as the patients’ age, tumor histology, molecular markers, and the extent of resection [17]. The absence of residual tumor after radical resection is one of the most significant prognostic factors in the progression-free survival of the affected patients [13, 19]. In recent times, the standardized use of 5-aminolevulinic acid has improved the extent of resection and thus contributed to the prolonged survival [19]. However, more radical surgical tumor removal is associated with a set of several complications. Amongst others, high-pressure tumor bed cysts showing a progressive, space-occupying growth pattern have been described as a possible complication [3, 11]. Such cysts occupy the former tumor bed and become symptomatic when leading to increased intracranial pressure. Although certain risk factors such as intraoperative opening of the ventricles, tumor malignancy, or previous radiotherapy have been identified, the pathogenesis of these cysts remains elusive. Several authors have proposed the formation of arachnoid slit valves as a possible underlying mechanism. Arachnoid slit valves are thought to act as a pressure valve, only allowing for unidirectional flow during peaks in intracranial pressure (ICP). In a recently published study, authors were able to observe in real time the dynamics of a slit valve mechanism as the underlying cause of space-occupying tumor bed cysts [4]. In the given case, the slit valve developed from a collagen fleece which was used to seal the opened lateral ventricle after HGG resection. However, slit valve mechanisms have also been discussed in the pathogenesis of enlarging primary arachnoid cysts [8, 14, 15]. The procedures that were employed in the treatment of slit valve–based tumor bed cysts and arachnoid cysts comprise excision of the valve, incision of the cyst wall, and cystoventricular and cystoperitoneal shunting, respectively [3, 8, 14, 22].

## Methods

**Fig. 1:**
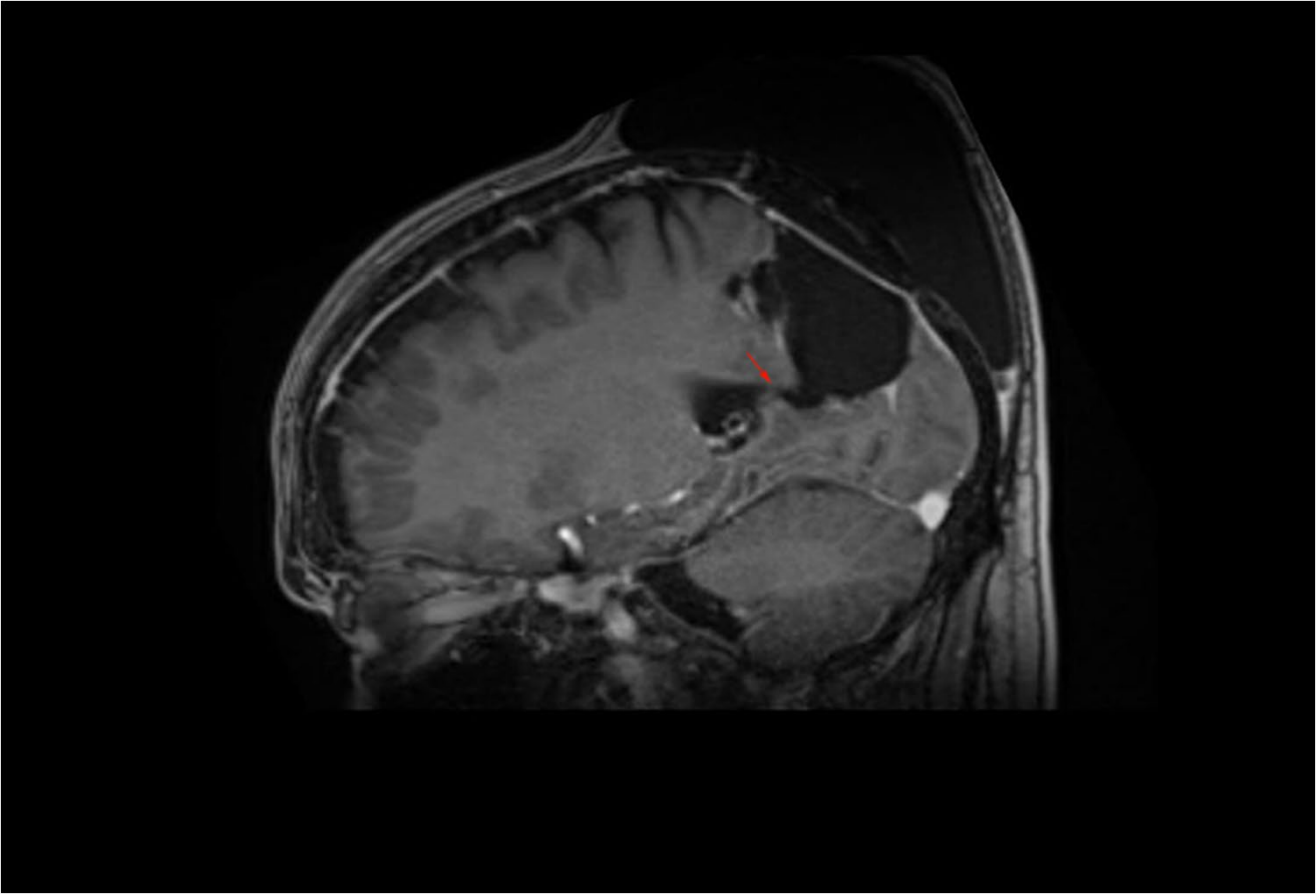
Preoperative sagittal T1-weighted MRI showing the tumor bed cyst and its communication with the epicranial CSF collection. Red arrow points to the location of the slit valve between the posterior horn of the lateral ventricle and the tumor bed

**Fig. 2:**
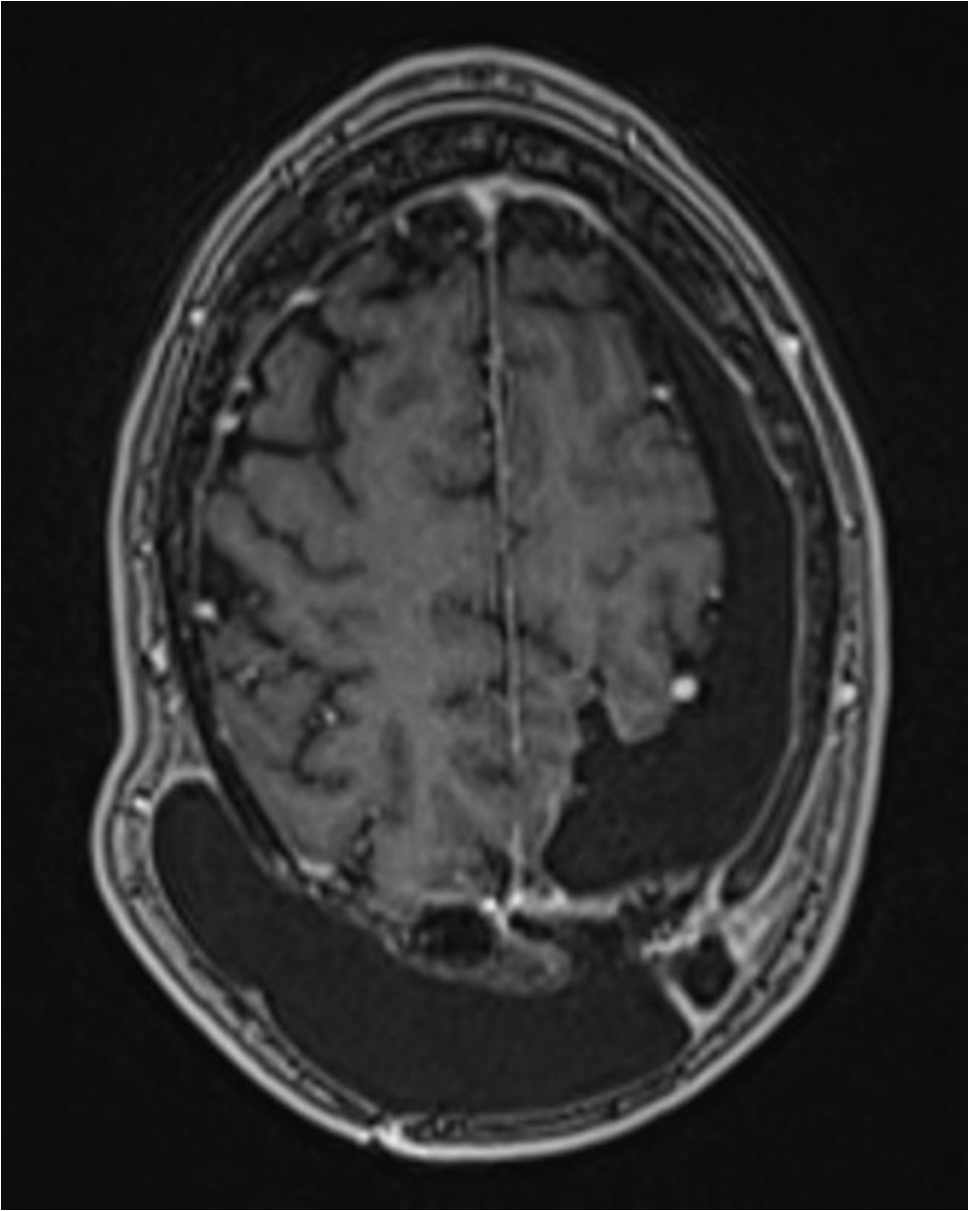
Preoperative axial T1-weighted MRI showing the extent of the subcutaneous CSF collection

In this study, we present the retrospective analysis of four patients treated by a novel surgical intervention for treating tumor bed cysts. All patients were treated in our university hospital between the years of 2010 and 2020. Patients were considered for the implantation of synthetic vascular grafts if they developed high-pressure tumor bed cysts following glioma resection. Elevated pressure in the tumor bed cysts was determined by clinical and radiographic findings that were indicative of pressure higher than that inside the physiological cerebrospinal fluid (CSF) spaces. Such findings comprised progressive cyst growth on consecutive imaging that extends over the boarders of the initial resection cavity; midline-shift and/or displacement of brain parenchyma/ ventricles; pericystic edema; major and progressive subcutaneous CSF collections (as depicted in Figs. 1 and 2); and clinical symptoms of elevated ICP such as somnolence or nausea and vomiting with no other underlying cause. In these cases, slit valves were considered the underlying pathomechanism. If a slit valve mechanism could then be observed intraoperatively after entering the cyst, the decision to implant the vascular graft into the lumen of the valve was finalized. We used 12-mm ring-walled GORE-TEX vascular graft (GORE-TEX Vascular Graft—Standard-walled Ringed, W. L. Gore & Associates, Inc., Newark, DE, USA) as a means of keeping the lumen of the slit valve open. The patients’ data are summarized and presented in Table 1. Preoperatively, all patients were informed about the off-label treatment and its novelty. All patients gave informed consent to the procedure. Postoperatively, all patients were monitored as inpatients and the success of the intervention was confirmed by cMRI 3 months postoperatively.

**Table 1:** Demographic data of each patient

Patient		1	2	3	4
Sex		m	m	f	m
Age		44	55	46	57
Tumor					
	WHO	IV	IV	III–IV	II–III
	Location	Parieto-occipital	Temporo-occipital	Frontal	Parieto-occipital
Months since implantation		124	49	20	8

## Results

Patient 1 is a 44-year-old man who was operated on a parieto-occipital glioblastoma (WHO IV) in 02/2010. As the tumor infiltrated the posterior horn of the left lateral ventricle, the ventricle was opened during the resection. Three weeks postoperatively, the patient presented to our department with symptoms of raised ICP. CCT imaging showed a space-occupying cyst in the former tumor bed causing significant midline-shift and perifocal edema. In a first endoscopic revision, a slit valve between the ventricle’s posterior horn and the cyst could be observed. Endoscopically, the wall separating the cyst and the ventricle was then fenestrated. However, postoperative control imaging soon revealed the persistence of the high-pressure cyst. In order to establish long-term communication between the tumor bed and the lateral ventricle, a 12-mm GORE-TEX vascular graft was implanted into the lumen of the slit valve as a means of keeping it open and establishing drainage. Revision-free survival following implantation of the synthetic vascular graft is 126 months for this patient.

Patient 2 is a 55-year-old male who underwent resection of a right temporo-occipital glioblastoma (WHO IV) in 08/2015 and re-resection in 01/16. Six months after removal of the tumor, the patient developed a tumor bed cyst that was initially treated by cystoperitoneal (CP) shunting. After three consecutive revisions of the shunt system due to blockage, the patient gave informed consent for the implantation of a GORE-TEX vascular graft. The implantation was conducted in a similar manner as was described for patient 1. After implantation, the CP shunt was left in situ. The patients’ symptoms are managed with both vascular graft and VP shunt and revision-free survival in this patient is 53 months.

Patient 3 is a 46-year-old woman who underwent resection of a left frontal anaplastic astrocytoma (WHO III) in 12/2014. After re-resection in 05/18, the histological diagnosis of the recurrent tumor was adjusted to a glioblastoma (WHO IV). Seven months after re-resection, the patient developed symptoms of elevated ICP and underwent cCT imaging. The cCT revealed a large high-pressure cyst occupying the former tumor bed. Intraoperatively, a slit valve between the tumor bed cyst and the frontal horn of the lateral ventricle could be observed. In this case, due to our experience with the recurrence rates of slit valve cysts when treated with fenestration or shunting, the patient was directly treated by implantation of a ring-walled GORE-TEX vascular graft. Following implantation of the vascular graft, the cyst was successfully managed for 10 months until the patient died in 09/19 due to tumor progression.

**Fig. 3:**
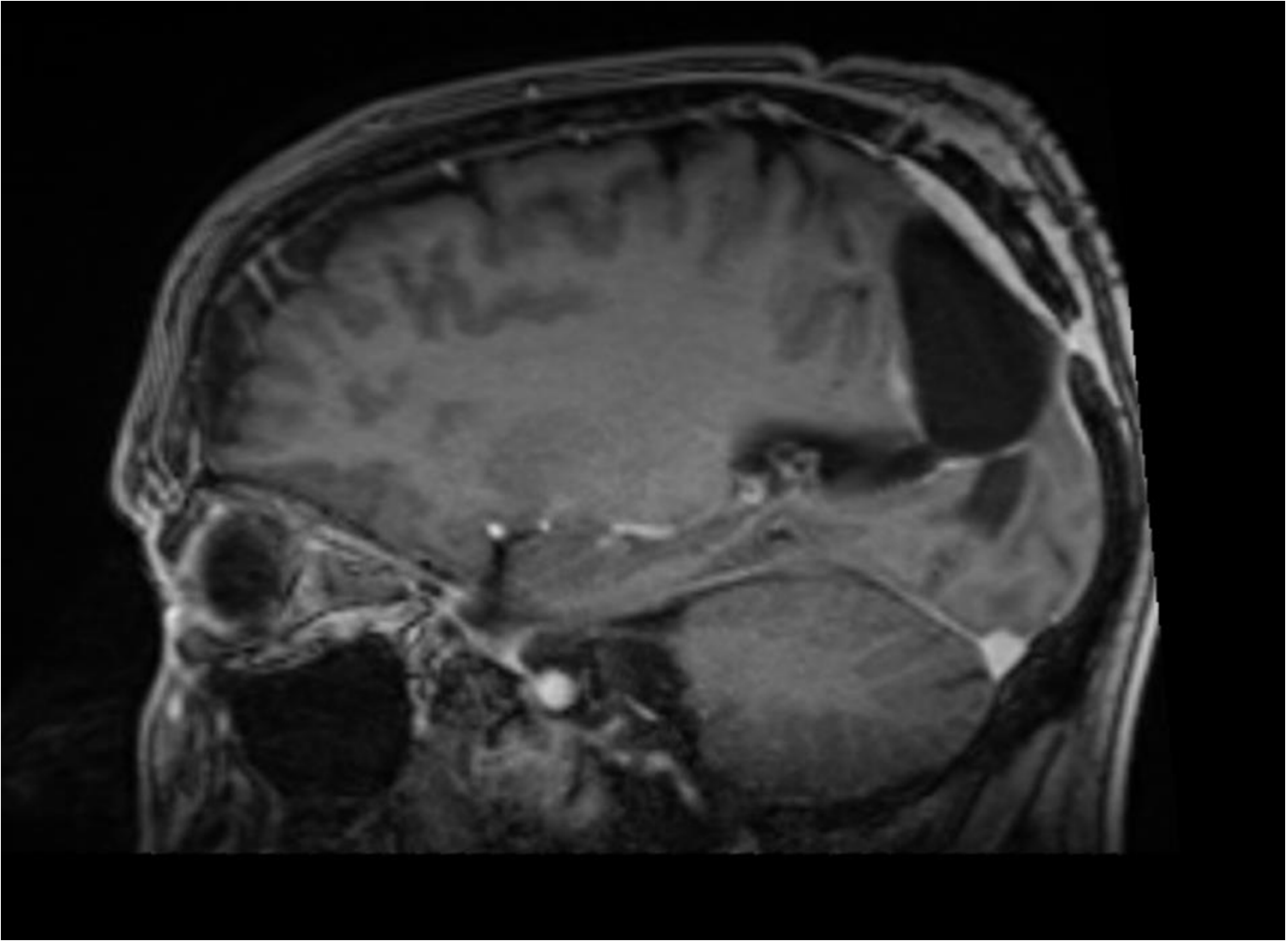
Postoperative sagittal T1-weighted MRI showing no residual subcutaneous CSF. Note the openly communicating tumor bed and posterior horn of the lateral ventricle

**Fig. 4:**
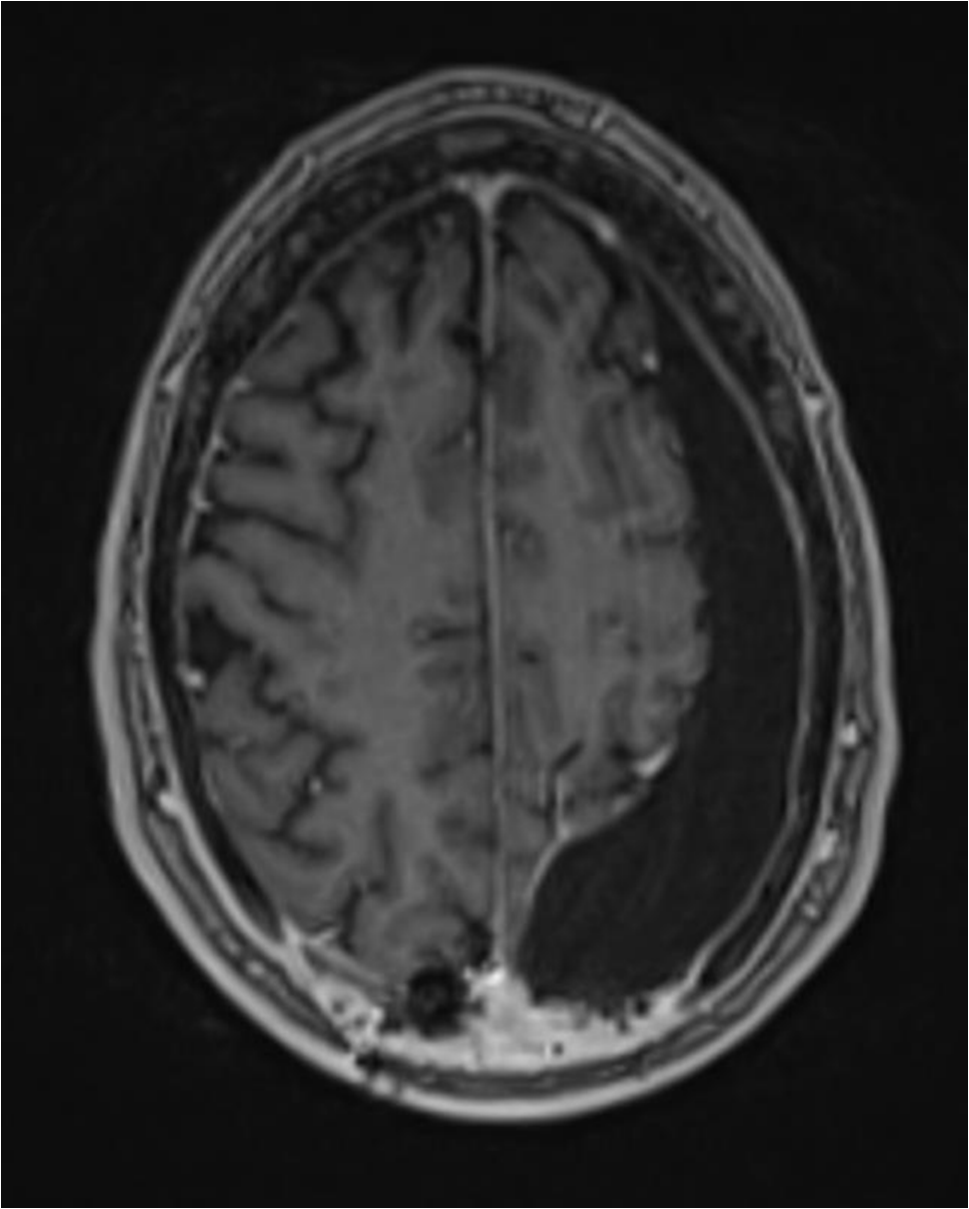
Postoperative axial T1-weighted MRI showing no residual subcutaneous CSF

**Fig. 5:**
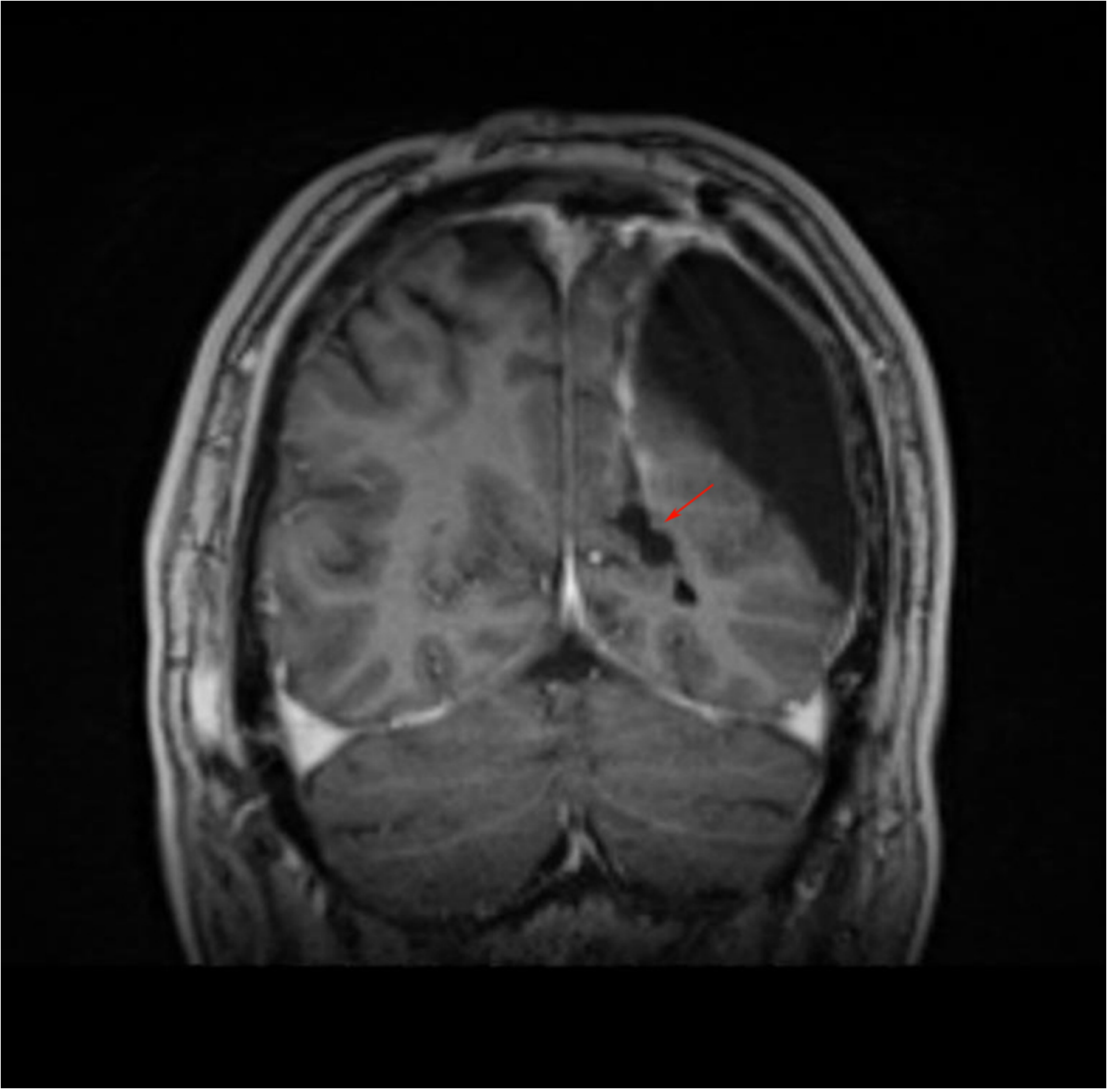
Postoperative coronal T1-weighted MRI. Red arrows point at the location where the circular outlet of the ringed vascular graft can be seen

**Fig. 6:**
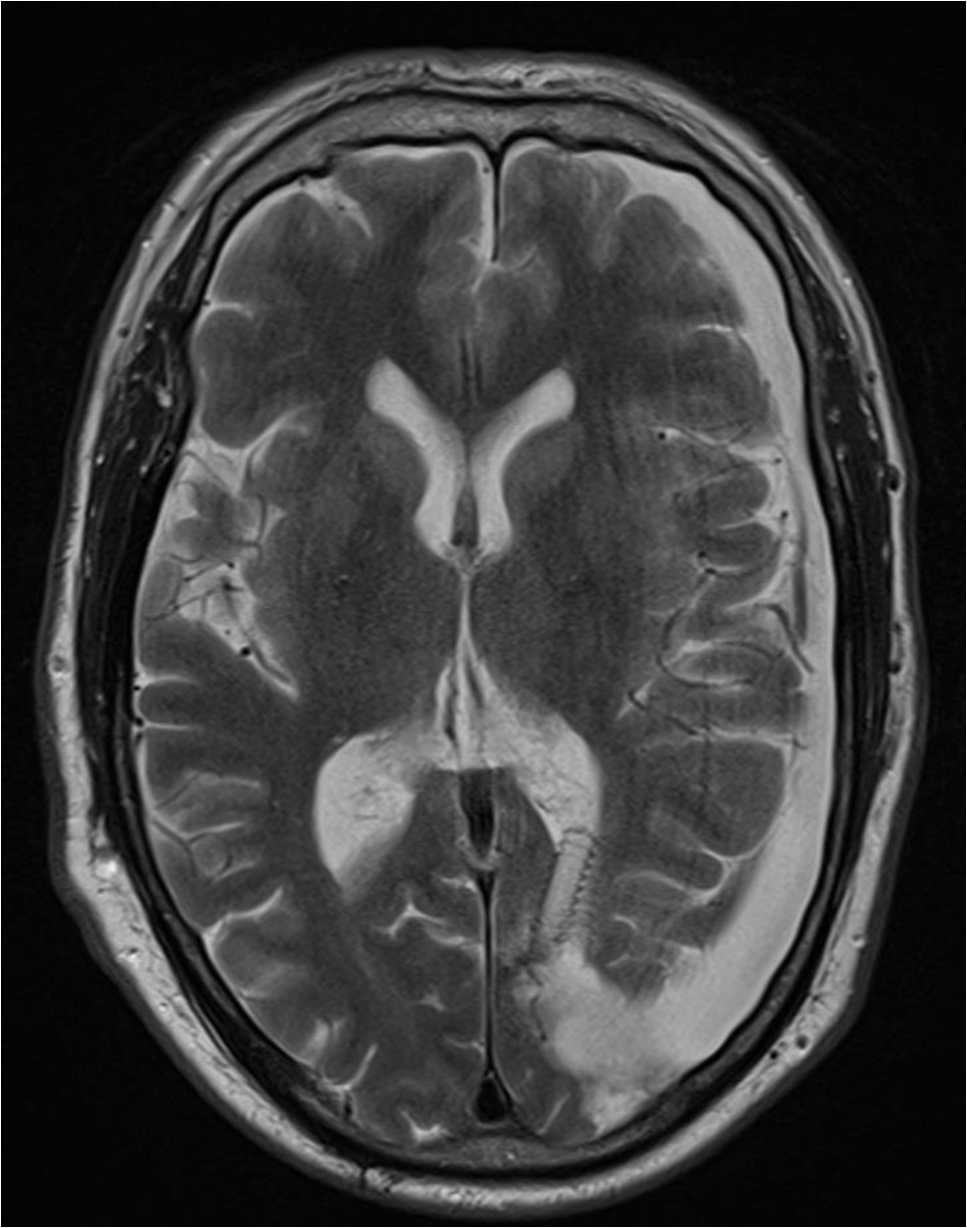
Postoperative SWI-weighted axial MRI showing the ringed wall structure of the vascular graft within the slit valve

Patient 4 is a 57-year-old male who was operated on a left parieto-occipital oligodendroglioma (WHO II) in 02/18. After resection of recurrent tumor mass in 10/19, the histological diagnosis was adjusted to an anaplastic glioma (WHO III). Following the second operation, the patient developed a sizeable subcutaneous collection of CSF. CMRI imaging revealed a tumor bed cyst that communicated with the subcutaneous CSF collection (the patient’s cMRI scans are depicted in Figs. 1 and 2). Other than in the previous cases, due to the drainage into the subcutaneous tissue, the tumor bed cysts did neither lead to midline-shift and perifocal edema nor to symptoms of elevated ICP. Initial trials of cyst puncture and tight head bandages proved unsuccessful so the patient underwent operative revision. Intraoperatively, after entering the former tumor bed, the entrance into the posterior horn of the left lateral ventricle was identified. At this point, we simulated increased ICP by executing a Valsalva maneuver which filled the former tumor bed with CSF from the posterior horn. Following the maneuver, the CSF got trapped and could not flow back in the opposite direction, reassuring the theory of a slit valve that allows for unidirectional flow. The same maneuver was conducted after the implantation of two 12-mm ring-walled GORE-TEX vascular grafts then showing unobstructed back-flow of the CSF. (The intraoperative video shows both the mechanism of tumor bed filling and the implantation of the synthetic ringed vascular grafts). Postoperatively, the CSF did not again recollect subcutaneously and the patient remains with the vascular graft until this day. The postoperative cMRI scans showing the reduction of the subcutaneous CSF and the vascular graft in situ are depicted in Figs. 3, 4, 5, and 6. Progression-free survival in this patient is 12 months.

## Discussion

The concept of slit valves as the underlying pathology of growing and potentially space-occupying cysts itself is not novel. Yet, it is neither routinely taken into consideration when making clinical decisions in these cases nor is it taught as a widely accepted pathomechanism. Several authors were able to clearly observe the mechanism both by means of cCT and cMRI imaging as well as in real time with an endoscope [4, 7, 10, 14, 15]. With our article demonstrating the slit valve mechanism during open brain surgery and simulating its dynamics via Valsalva maneuver, this evidence is further supported. Considering all data on the phenomenon of iatrogenic slit valves, the authors believe that this concept is sufficiently evidenced in order to merit consideration whenever patients develop high-pressure space-occupying tumor bed cysts following tumor resection.

In our department, the implantation of vascular grafts was invented out of a lack of efficient traditional treatments. As described in the individual patients, other treatment options such as fenestration or CP shunting are associated with high complication and recurrence rates. Shunt malfunctioning in CP shunts is a common adverse effect [6, 18]. Considering the fact that the cystic fluid is often not only normal CSF but richer in its content of proteins, the rate of shunt malfunctions in these cases might be higher compared to cases of ventriculoperitoneal (VP) shunting for hydrocephalus—although works systematically evaluating this possibility are still lacking [5]. Another thing that must be taken into consideration when evaluating a patient with a tumor bed cyst for CP shunting is their limited life expectancy [12, 17]. On the long term, the complications of a CP shunt are likely to increase the total time of hospitalization for the affected patients and thereby decrease their quality of life [1]. In our group of patients, none of the implanted vascular grafts was marked by postoperative complications. While a bigger cohort and a longer follow-up is needed to objectively control this technique for complications, the fact that the grafts do not connect two otherwise separated compartments will be an advantage in the prevention of infections compared to CP shunts.

All things considered, the authors deem it reasonable to reevaluate the management of iatrogenic space-occupying cysts. In any postoperative case with such cyst development, the eventuality of an underlying slit valve mechanism should be investigated. Our experience has shown that the risk for slit valves seems to be increased when tumor beds communicate with an opening into the posterior or anterior horn of the lateral ventricle. In these cases, the slit valve formation took place at the smallest point of outlet. Other works have shown that repairing of openings in the ventricle wall with collagen fleece can also be considered a possible risk factor [4]. In the reported case, the fleece itself was still found in situ and with a central tear which was functioning as the slit valve.

As mentioned before, the herein presented use of GORE-TEX vascular grafts was conducted in an off-label setting of compassionate care. We deemed its use reasonable and safe based on several considerations. Firstly, GORE-TEX is a fabric membrane composed of physically expanded polytetrafluoroethylene (PTFE, generically known as Teflon). A multitude of medical devices made out of PTFE/Teflon have been approved for medical use by the FDA. Therefore, we considered its use safe for this study. In addition to that, GORE-TEX dura substitutes (Gore Preclude MVP Dura Substitute, W. L. Gore & Associates, Inc., Newark, DE, USA) consisting of the same fabric are FDA-approved for neurosurgical interventions and enjoy widespread use in dural plasty or replacement [2, 16, 21].

This study has several limitations. We were only able to retrospectively report the clinical history of four patients that have been treated with this novel procedure in an off-label and compassionate use setting. In order to properly evaluate the procedure, prospective studies with a bigger quantity of patients must be conducted. Moreover, the details of the technique remain purely experimental. Points such as the sort of vascular graft used, having it fixed or just laid in or long-term complications, must be addressed in future studies.

## Conclusion

In conclusion, we believe that the evidence published on slit valves is sufficient to establish their existence as a possible pathomechanism of high-pressure cyst formation. In case of cyst formation in such a manner, we deem the use of synthetic ringed vascular grafts as a means of stenting the iatrogenic slit valve a promising new treatment option.

## Supplementary information

ESM 1 Video The intraoperative video shows how recommunication of the tumor bed and ventricular system was established by implanting two synthetic ringed vascular grafts. The mechanism tumor bed filling due to a slit valve is demonstrated by use of the Valsalva maneuver (MP4 49478 kb)

## Data Availability

All data that has been evaluated for this study is presented in the manuscript.

## Declarations

All research presented in this manuscript has been conducted in accordance with the ethical standards laid down in the 1964 Declaration of Helsinki and its later amendments. The off-label use of the vascular grafts as presented in the article has been conducted in accordance with the local German law regarding the compassionate use of off-label indications. Therefore, an IRB/ ethic committee approval was not obtained.
